# Supplementary material for: Association between oral microbiome and seven types of cancers in East Asian population: a two-sample Mendelian randomization analysis
Source: Front Mol Biosci. 2023 Nov 21;10:1327893. doi: 10.3389/fmolb.2023.1327893 (PMC10702768; doi:10.3389/fmolb.2023.1327893)
Supplement: Supplementary file 1 [file DataSheet1.ZIP › Supplementary Materials/MR plots for tongue/tongue═╝/Colorectal cancer/pheno.866_to_colorectal cancer_leave_one_out.pdf]

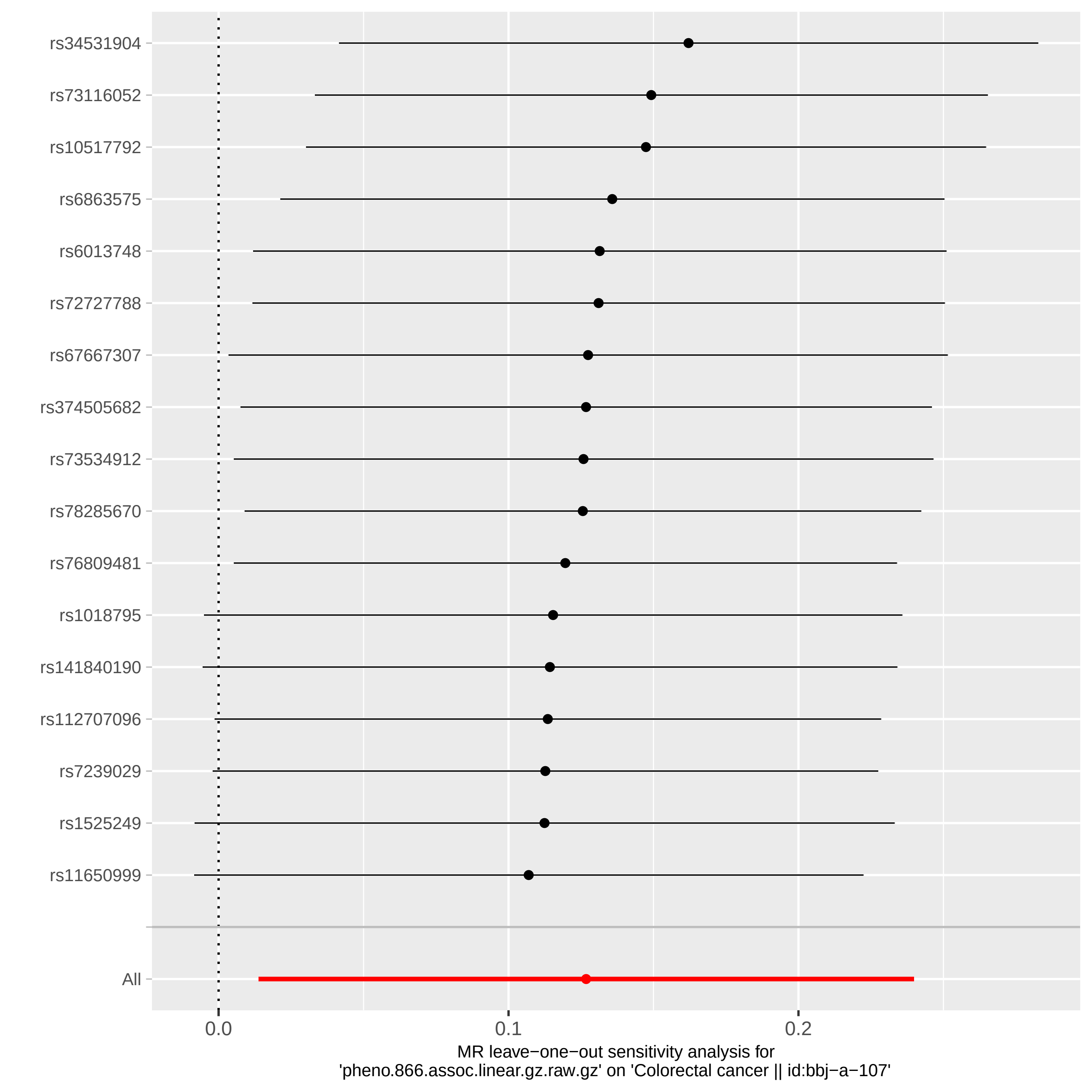
rs34531904
rs73116052
rs10517792
rs6863575
rs6013748
rs72727788
rs67667307
rs374505682
rs73534912
rs78285670
rs76809481
rs1018795
rs141840190
rs112707096
rs7239029
rs1525249
rs11650999
All
0.0
0.1
0.2
MR leave−one−out sensitivity analysis for
'pheno.866.assoc.linear.gz.raw.gz' on 'Colorectal cancer || id:bbj−a−107'
